# Calcium Spark Detection and Event-Based Classification of Single Cardiomyocyte Using Deep Learning

**DOI:** 10.3389/fphys.2021.770051

**Published:** 2021-11-08

**Authors:** Shengqi Yang, Ran Li, Jiliang Chen, Zhen Li, Zhangqin Huang, Wenjun Xie

**Affiliations:** ^1^Beijing Engineering Research Center for IoT Software and Systems, Beijing University of Technology, Beijing, China; ^2^The Key Laboratory of Biomedical Information Engineering of Ministry of Education, Institute of Health and Rehabilitation Science, School of Life Science and Technology, Xi’an Jiaotong University, Xi’an, China

**Keywords:** Ca^2+^ sparks, deep learning, automated detection, classifying single cardiomyocyte, cardiac diseases

## Abstract

Ca^2+^ sparks are the elementary Ca^2+^ release events in cardiomyocytes, altered properties of which lead to impaired Ca^2+^ handling and finally contribute to cardiac pathology under various diseases. Despite increasing use of machine-learning algorithms in deciphering the content of biological and medical data, Ca^2+^ spark images and data are yet to be deeply learnt and analyzed. In the present study, we developed a deep residual convolutional neural network method to detect Ca^2+^ sparks. Compared to traditional detection methods with arbitrarily defined thresholds to distinguish signals from noises, our new method detected more Ca^2+^ sparks with lower amplitudes but similar spatiotemporal distributions, thereby indicating that our new algorithm detected many very weak events that are usually omitted when using traditional detection methods. Furthermore, we proposed an event-based logistic regression and binary classification model to classify single cardiomyocytes using Ca^2+^ spark characteristics, which to date have generally been used only for simple statistical analyses and comparison between normal and diseased groups. Using this new detection algorithm and classification model, we succeeded in distinguishing wild type (WT) vs RyR2-R2474S^±^ cardiomyocytes with 100% accuracy, and vehicle vs isoprenaline-insulted WT cardiomyocytes with 95.6% accuracy. The model can be extended to judge whether a small number of cardiomyocytes (and so the whole heart) are under a specific cardiac disease. Thus, this study provides a novel and powerful approach for the research and application of calcium signaling in cardiac diseases.

## Introduction

Since the discovery of Ca^2+^ sparks in 1993, this elementary sarcoplasmic reticulum (SR) Ca^2+^ release event in cardiomyocytes has attracted enormous attention (Cheng et al., 1993; Cheng and Lederer, 2008). A typical Ca^2+^ spark has small spatial size (∼2 μm) and fast kinetics (∼20 ms) (Cheng and Lederer, 2008), and high-resolution line-scan imaging is required to accommodate such spatiotemporally defined fluorescent events as Ca^2+^ sparks, thereby resulting in noisy images (Cheng and Lederer, 2008). To date, several tools have been developed to detect Ca^2+^ sparks in line-scan Ca^2+^ images, most of which introduced an arbitrary threshold comprising the mean intensity μ and background standard deviation σ, usually μ+3.8σ, to distinguish the tiny signals from noises (Cheng et al., 1999; Wegner et al., 2006; Picht et al., 2007; Steele and Steele, 2014). A connected problem was that of “undetected sparks,” i.e., smaller fluorescence signals below the arbitrarily defined threshold (Cheng et al., 1999), which may lead to inaccurate estimates of spark probability, average spark flux, and other parameters, especially under some cardiac diseases for which the average amplitude of Ca^2+^ sparks in cardiomyocytes could be significantly reduced (Cheng and Lederer, 2008; Brochet et al., 2011; Shan et al., 2012; Santulli et al., 2015).

Excitation-contraction coupling (ECC) converts electrical stimuli to mechanical forces in cardiomyocytes. Impaired ECC are closed associated with cardiac pathology (Kansakar et al., 2021). ECC starts with the minimal influx of Ca^2+^ *via* the L-type Ca^2+^ channels, which triggers SR Ca^2+^ release by activating type 2 ryanodine receptor Ca^2+^ release channel (RyR2) (Cheng and Lederer, 2008; Kansakar et al., 2021). A number of reports have revealed altered SR Ca^2+^ handling in various cardiac diseases such as atrial fibrillation, heart failure (HF), and catecholaminergic polymorphic ventricular tachycardia (CPVT)—as the cause or consequence of cardiac dysfunction (Shan et al., 2012; Santulli et al., 2015; Xie et al., 2015; Zhang et al., 2021). As elementary SR Ca^2+^ release events in cardiomyocytes, Ca^2+^ sparks were widely used as readouts to distinguish normal and diseased cardiomyocytes (Shan et al., 2012; Santulli et al., 2015; Xie et al., 2015; Huang et al., 2021; Kansakar et al., 2021; Zhang et al., 2021). While most of those studies simply used the mean values of one or a few characteristics of Ca^2+^ sparks—including frequency, amplitude, and spatiotemporal parameters—for statistical significance comparison between normal and diseased groups, whereas attempts at deep digging and how to use these learnt data have rarely been reported.

With recent advances in machine learning and deep learning, a collection of algorithms with impressive abilities to decipher the content of images is now available to researchers in medical and biological fields (Esteva et al., 2017; Zhang et al., 2019). Machine learning builds a mathematical model based on structured training data to make predictions or decisions on new data. Deep learning is a subset of machine learning with supervised and unsupervised approaches. It gained attention when a deep-learning-based method won the 2012 ImageNet Large Scale Visual Recognition Challenge. Since then, there has been a major increase in the variety of problems that can be solved with deep learning. In addition, improvements in computer hardware and deep-learning frameworks have placed these tools within reach of the typical software developer, and they are becoming increasingly popular in bio/medical fields. Because deep-learning algorithms can distinguish objects with different characteristics, a deeper processing and analyzing Ca^2+^ spark images is expected.

In this study, we proposed a deep-learning framework for automated Ca^2+^ spark detection, analysis, and event-based classification of single cardiomyocytes, including the following. (1) To better characterize Ca^2+^ sparks in cardiomyocytes under diseased conditions, we developed a deep-learning method for automated spark detection that is free of arbitrarily defined thresholds and can detect a greater number of weak signals. (2) Based on the characteristics of Ca^2+^ sparks detected above, we proposed an event-based binary classification model to identify single cardiomyocytes into normal or diseased groups with promising accuracy.

## Methods

### Animal

All experimental protocols were approved by the Institutional Animal Care and Use Committee of Xi’an Jiaotong University and conformed to the Guide for the Care and Use of Laboratory Animals published by the National Institutes of Health. Five-month-old male mice harboring CPVT mutation RyR2-R2474S^±^ (RS) (Shan et al., 2012; Xie et al., 2013) or wild-type littermates (WT) were anesthetized with an intraperitoneal injection of sodium pentobarbital (80 mg/kg) and then euthanized by cervical dislocation.

### Data Acquisition

Adult murine cardiomyocytes were isolated and subjected to line-scan Ca^2+^ imaging as described previously (Xie et al., 2015; Huang et al., 2021; Zhang et al., 2021). For Ca^2+^ spark measurement, cardiomyocytes were preloaded with 5-μmol/L fluo-4 AM (Thermofisher) for 15 min and subjected to line-scan imaging at a speed of 400 lines/s on a Leica TCS SP8 confocal microscope with 40× magnification and a 1.3-NA oil immersion objective. The scan zoom was adjusted to fit the cells, and the scan line was along their long axis. The excitation for Fluo-4 was 488 nm, and emission was collected at 505–530 nm. Ca^2+^ spark detection and analysis were performed using either the traditional method (Cheng et al., 1999) or the algorithms described in the Results section.

### Deep Learning Method Architecture

**FIGURE 1:**
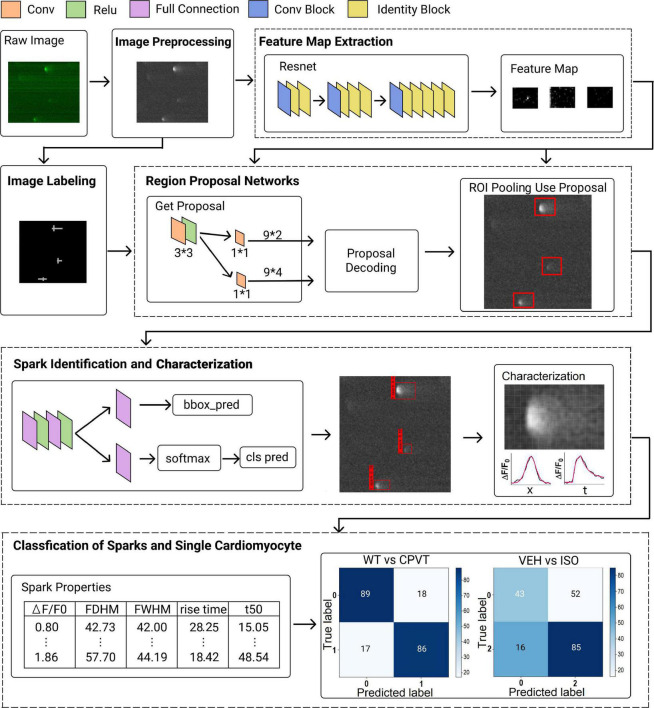
Network architecture of proposed framework. The framework comprises several major processes, including image pre-processing and labeling, feature map extraction, region proposal network, spark identification and characterization, and classification of sparks and single cardiomyocytes.

The deep learning method architecture is composed of several major processes, including image pre-processing and labeling, feature map extraction, region proposal network, spark classification and characterization, and classification of sparks and single cardiomyocytes (Figure 1). A detailed description of each process please refers to the Supplementary materials.

### Statistics

Data are reported as mean ± standard error. Student’s *t*-test (for two groups) or one-way ANOVA with Tukey’s multiple comparison test (for three or more groups) was carried out using GraphPad Prism (version 6.00, GraphPad Software, La Jolla, CA, United States). Differences were considered statistically significant at *P* < 0.05.

## Results

**FIGURE 2:**
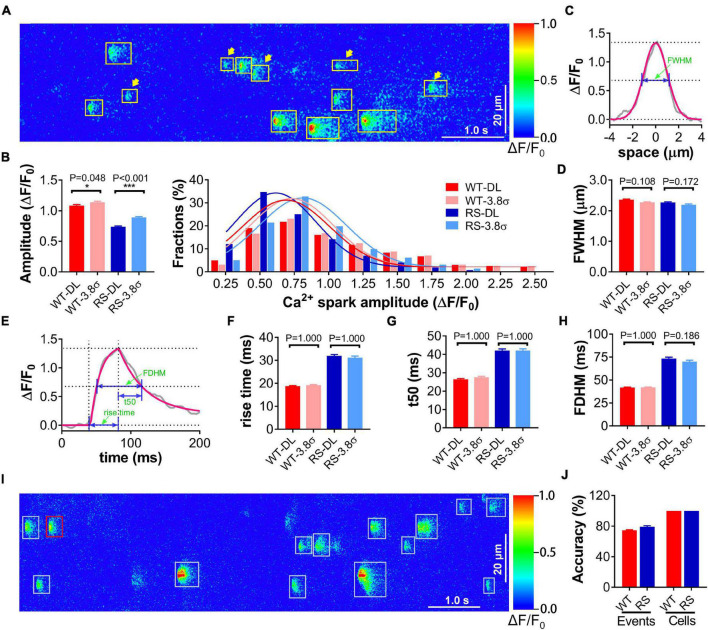
Application of proposed method for spark detection and event-based classification of single cardiomyocytes using Ca^2+^ spark characteristics. **(A)** An example of Ca^2+^ sparks in a line-scan image of an RS cardiomyocyte detected by deep-learning methods. All detected sparks are labeled with rectangles, and the arrowheads indicate low-amplitude events (peak *F*/*F*_0_ < μ + 3.8σ). **(B)** Statistics and distributions of Ca^2+^ spark amplitude in WT and RS cardiomyocytes using conventional (3.8σ) and deep learning (DL) method. **(C)** Raw (gray)/Gaussian fitting (red) spatial traces of a representative Ca^2+^ spark. FWHM is calculated from the fitting curve as illustrated. **(D)** Statistics of FWHM of Ca^2+^ sparks in each group. **(E)** Raw (black)/fitting (Lacampagne et al., 1999) (red) time courses of a representative Ca^2+^ spark. Rise time, *t*50, and FDHM are calculated from the fitting curve as illustrated. **(F–H)** Statistics of temporal parameters of Ca^2+^ sparks in each group. *n* = 1353 (DL)/1281 (3.8σ) or 1061 (DL)/807 (3.8σ) events in 108 WT or 52 RS cardiomyocytes. **(I)** An example of spark classification in a line-scan Ca^2+^ image of an RS cardiomyocyte and **(J)** statistics of event- and cell-classification accuracy in the independent test dataset comprising 46 WT and 30 RS cardiomyocytes, respectively. Each spark is first classified as being in the “WT” (red) or “RS” (cyan) group, then a cardiomyocyte with more (resp. fewer) “WT” events is classified as a “WT” (resp. “RS”) one.

We performed the test for spark detection in cardiomyocytes from RS and WT mice using the proposed deep-learning method with the traditional method (Cheng et al., 1999) as the control. RyR2-R2474S is a typical human CPVT-linked RyR2 mutation. Previous studies have shown that cardiomyocytes from RS mice displayed obviously increased frequency and reduced amplitude of Ca^2+^ sparks compared to the WT group (Shan et al., 2012; Xie et al., 2013, 2015). The training data set included 3611 Ca^2+^ sparks from line-scan Ca^2+^ images of 100 WT and 100 RS cardiomyocytes. The test data set comprised line-scan Ca^2+^ images from 108 WT and 52 RS cardiomyocytes. Many signals with low peak *F*/*F*_0_ (<3.8σ) that were omitted by the traditional method were detected by the proposed deep-learning method, especially in RS cardiomyocytes (Figure 2A), resulting in a statistically lower amplitude of events (Figure 2B), while the spatiotemporal characteristics remained similar between both methods, indicating that these low-amplitude events were also Ca^2+^ sparks and not noises (Figures 2C–H).

Furthermore, we attempted to classify single cardiomyocytes as “WT” or “RS” using the logistic regression and classification model trained with the characteristics of Ca^2+^ sparks detected above. In the independent test dataset including 46 WT and 30 RS cardiomyocytes, 74.71% of the “WT” and 79.19% of the “RS” Ca^2+^ sparks were classified correctly (Figures 2I,J). The lowest event classification accuracy in single cardiomyocytes was 65.0 or 64.3% for the WT or RS group, respectively. Thus, according to the major event classification in each cardiomyocyte, the accuracy for cell classification into the WT and RS groups is 100% (Figures 2I,J).

**FIGURE 3:**
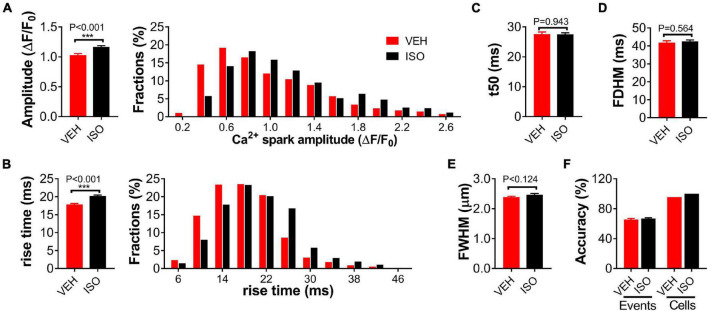
Classification of single WT cardiomyocytes treated with isoprenaline or vehicle using Ca^2+^ spark characteristics. Statistics and distributions of amplitude **(A)** and rise time **(B)** of Ca^2+^ sparks in WT cardiomyocytes treated with 0.1-μmol/L isoprenaline (ISO) or vehicle (VEH). These parameters show obviously different distributions between the two groups. Statistics of *t*50 **(C)**, FDHM **(D)**, and FWHM **(E)** display no difference. *n* = 557 or 675 events from ≥20 cardiomyocytes per group. **(F)** Statistics of event and cell classification accuracy in the independent test dataset comprising 46 “VEH” and 20 “ISO” treated WT cardiomyocytes from at least five mice.

Compared to specific genetic mutation, overactivation of the sympatho-β-adrenergic receptors (β-ARs) system is a common and important mechanism of various cardiac diseases. *In vitro* application of isoprenaline is a typical model mimicking β-AR stimulation in cardiomyocytes. We also tested the effectiveness of the present method in classifying cardiomyocytes under 0.1-μmol/L isoprenaline (ISO) stimulation or vehicle (VEH) treatment. The amplitude and rise time of the detected Ca^2+^ sparks differed significantly between the ISO and VEH groups, while the other parameters were similar (Figures 3A–E). Our method achieved 65.51 and 66.81% accuracy for event classification, and 97.8 and 100% accuracy for cardiomyocyte classification, respectively (Figure 3F).

## Discussion

As elementary SR Ca^2+^ release events in cardiomyocytes, Ca^2+^ sparks were widely used as readouts to distinguish normal and diseased cardiomyocytes (Shan et al., 2012; Santulli et al., 2015; Xie et al., 2015; Huang et al., 2021; Kansakar et al., 2021; Zhang et al., 2021). Despite various alterations of Ca^2+^ spark characteristics in diverse diseases, in most cases only simple statistical analyses and comparison have been performed for using Ca^2+^ sparks (e.g., as a readout for diastolic SR Ca^2+^ leakage), whereas attempts at deep digging and how to use these learnt data have rarely been reported. In the present study, we proposed an event-based logistic regression and binary classification model to classify single cardiomyocytes using Ca^2+^ spark characteristics. With deep learning, our classification model succeeded in distinguishing WT and CPVT-mutated cardiomyocytes with 100% accuracy, and it distinguished isoprenaline-insulted WT cardiomyocytes from vehicle ones with 95.6% accuracy. Chronic overactivation of the β-AR system is a common and important mechanism of various cardiac diseases (e.g., HF). Therefore, the proposed classification model should work well to identify whether a heart is under a specific disease or quantify the disease development using Ca^2+^ spark images of a small number of cardiomyocytes, through presetting the classification model with Ca^2+^ spark properties of cardiomyocytes under normal and the specific diseased conditions.

Because Ca^2+^ sparks are events with small spatial size and fast kinetics, traditional methods have usually introduced an arbitrarily defined threshold (μ+3.8σ) to exclude noises from the true tiny signals in the confocal line-scan Ca^2+^ images. However, because of the differences in opening times and channels of local Ca^2+^ release units, the SR Ca^2+^ content, and the distance between focal plane and release sites, the amplitudes of Ca^2+^ sparks vary widely. Thus, cutoff with a threshold has resulted in omission of weak but true Ca^2+^ sparks, while lowering the threshold can lead to false positive detection. The missed and false-positive events can distort the distribution of measured Ca^2+^ spark properties. Despite continuous progression in confocal microscopy, fluorescent indicators, and denoising algorithms in recent decades that have greatly improved the SNR of line-scan Ca^2+^ images, the detection problem awaits further resolution, especially under diseased conditions with many Ca^2+^ sparks in cardiomyocytes. The deep-learning method proposed in this study is free of any hard threshold for better detecting low-amplitude spark events and is shown to be more accurate for characterizing the difference of Ca^2+^ sparks between normal and diseased cardiomyocytes.

As how to detect the low-amplitude Ca^2+^ sparks remains to be solved for this fields, the labeling process of Ca^2+^ sparks in the training set is done manually with the assistance of a spark detection and characterization tool set to exclude false positive events, which could not guarantee that all low-amplitude events in the training images have been included and might lead to false negative detection. However, with these labeled low-amplitude events in the training set, our network can detect many more low-amplitude events in the test set, which is expected to improve further toward the final resolution of detecting low-amplitude Ca^2+^ sparks by self-adaption training procedure. Our training set included experimental line-scan Ca^2+^ spark images with a signal-to-noise ratio (SNR) of between ∼1.75 and ∼3.30, thereby allowing our model to detect spark events from images with SNR values in the same range. As our proposed approach is currently much slower than the traditional threshold-based algorithms, a further optimization in our algorithms will be required.

Taken together, the present study provides a novel and powerful approach for the research and application of calcium signaling in diseased hearts.

## Data Availability Statement

The datasets presented in this study can be found in online repositories. The names of the repository/repositories and accession number(s) can be found below: https://github.com/BJUT-XJTU-DigitalCell/Software.

## Ethics Statement

The animal study was reviewed and approved by Institutional Animal Care and Use Committee of Xi’an Jiaotong University.

## Author Contributions

SY and WX designed the research. SY, RL, JC, ZL, and WX performed the research. SY, RL, JC, and WX analyzed the data. SY, ZH, and WX wrote the manuscript. All authors contributed to the article and approved the submitted version.

## Conflict of Interest

The authors declare that the research was conducted in the absence of any commercial or financial relationships that could be construed as a potential conflict of interest.

## Publisher’s Note

All claims expressed in this article are solely those of the authors and do not necessarily represent those of their affiliated organizations, or those of the publisher, the editors and the reviewers. Any product that may be evaluated in this article, or claim that may be made by its manufacturer, is not guaranteed or endorsed by the publisher.
