# Supplementary material for: Calcium Spark Detection and Event-Based Classification of Single Cardiomyocyte Using Deep Learning
Source: Front Physiol. 2021 Nov 8;12:770051. doi: 10.3389/fphys.2021.770051 (PMC8607692; doi:10.3389/fphys.2021.770051)
Supplement: Supplementary file 1 [file Data_Sheet_1.pdf]

Calcium Spark Detection and Event-based Classification of Single Cardiomyocyte Using Deep Learning

RUNNING TITLE: Deep learning of calcium sparks

Shengqi Yang, Ran Li, Jiliang Chen, Zhen Li, Zhangqin Huang, Wenjun Xie

Supplementary methods

Preprocessing and Labeling

We selected 100 RS and 100 WT experimental line-scan Ca^{2+} images (resolution 512×9000) for the network training. After removing data points at extremes using a 5×5 median filter and normalization by using the algorithms as described previously (1), each line-scan Ca^{2+} image was cropped into 512×600 sub-resolution images and labeled to mark the spark locations. This spark labeling was done manually with the assistance of a spark detection and characterization tool set in according to the algorithms as described previously (1). Once all the high-amplitude labeled events had been selected automatically using the tools, the low-amplitude events were labeled manually and assessed with the tools to guarantee that all the labeled events were true sparks. Thus, although not all low-amplitude events were labeled in the training set, this approach helped to detect many more low-amplitude events (Supple. Fig. 1). In total, 3611 Ca^{2+} sparks were labeled for training. For each spark, the labeling algorithm positioned its center and took one line of spatial pixels (15-100 pixels depending on the spatial size of the spark) and one line of temporal pixels (15-100 pixels depending on the duration of the spark) to mark its position. The resulting labeled image contained many white crosses on a dark background to indicate the central energy and position of each spark.

Feature Map Extraction

The proposed method is based on the Faster-RCNN architecture, with feature map extraction using ResNet (2, 3). Faster-RCNN was extended from Fast RCNN (2-4) and was further extended to Mask RCNN for precise segmentation (5). Another well-known segmentation tool for medical and biological images is U-Net (6). U-Net is normally referred to as one-stage semantic segmentation, and Mask RCNN is a two-stage instance segmentation for more-precise

object segmentation. In the present work, the focus was on detecting a region of interest (ROI) and then characterizing it accurately, and so Faster-RCNN was used. Deep residual networks show promising accuracy gains from greatly increased depth with reduced training error and easy depth optimization. ResNet brings the output of a given layer directly to one specific subsequent layer as a linear contributor and performs feature map extraction. ResNet's multi-layer design is built with two basic blocks: *conv_block* and *identity_block*. The input and output dimensions of *conv_block* are different, thereby changing the dimension of the network; those of *identity_block* are the same, thereby deepening the network. Both *conv_block* and *identity_block* have two data paths as shown in Supple. Fig. 2A. In this figure, Conv2d is a general two-dimensional convolution operation; BatchNorm is batch normalization used to accelerate deep network training by reducing internal covariate shift (7); ReLu is a rectified linear unit, which is used widely in CNN to help prevent exponential growth in the computation required to operate the neural network; zeropad is when a border of pixels all with value zero is added around the edge of an image, thereby allowing the input image size to be adjusted; MaxPool is a common down-sampling strategy in CNN, and its output is a feature map containing the most prominent features of the input feature map. The left-side data path comprises three layers of Conv2d, BatchNorm, and ReLu operations. The right side is the data bypass path. At the third layer, before the ReLu operation, *conv_block* adds the convoluted input, while *identity_block* adds the bypassed input on top of the output of BatchNorm. In the present work, the input size was 600×600×3, and as a result of this process 38×38×1024 feature maps were produced for the next stage. ResNet produces five different decreased output sizes and the final output is the feature map, used in the ROI. Typical ResNet networks with different layers (18, 34, 50, 101, and 152 layers) are shown in Supple. Table 1. We compared three different layer choices—ResNet34, ResNet50, and ResNet101—in terms of execution time, loss, and accuracy (Supple. Fig. 2B–D). Based on these performances, ResNet50 was chosen for the subsequent experiments.

Supplementary Table 1. ResNet network with different layers for input image size of 600×600×3

layer name	output size	18-layer	34-layer	50-layer	101-layer	152-layer
conv1	112×112	7×7, 64, stride 2				
conv2_x	56×56	3×3 max poll, stride 2				
		$\begin{bmatrix}3\times3,64\\3\times3,64\end{bmatrix}\times2$	$\begin{bmatrix}3\times3,64\\3\times3,64\end{bmatrix}\times3$	$\begin{bmatrix}1\times1,64\\3\times3,64\\1\times1,256\end{bmatrix}\times3$	$\begin{bmatrix}1\times1,64\\3\times3,64\\1\times1,256\end{bmatrix}\times3$	$\begin{bmatrix}1\times1,64\\3\times3,64\\1\times1,256\end{bmatrix}\times3$
conv3_x	28×28	$\begin{bmatrix}3\times3,128\\3\times3,128\end{bmatrix}\times2$	$\begin{bmatrix}3\times3,128\\3\times3,128\end{bmatrix}\times4$	$\begin{bmatrix}1\times1,128\\3\times3,128\\1\times1,512\end{bmatrix}\times4$	$\begin{bmatrix}1\times1,128\\3\times3,128\\1\times1,512\end{bmatrix}\times4$	$\begin{bmatrix}1\times1,128\\3\times3,128\\1\times1,512\end{bmatrix}\times8$
conv4_x	14×14	$\begin{bmatrix}3\times3,256\\3\times3,256\end{bmatrix}\times2$	$\begin{bmatrix}3\times3,256\\3\times3,256\end{bmatrix}\times6$	$\begin{bmatrix}1\times1,256\\3\times3,256\\1\times1,1024\end{bmatrix}\times6$	$\begin{bmatrix}1\times1,256\\3\times3,256\\1\times1,1024\end{bmatrix}\times23$	$\begin{bmatrix}1\times1,256\\3\times3,256\\1\times1,1024\end{bmatrix}\times36$
conv5_x	7×7	$\begin{bmatrix}3\times3,512\\3\times3,512\end{bmatrix}\times2$	$\begin{bmatrix}3\times3,512\\3\times3,512\end{bmatrix}\times3$	$\begin{bmatrix}1\times1,512\\3\times3,512\\1\times1,2048\end{bmatrix}\times3$	$\begin{bmatrix}1\times1,512\\3\times3,512\\1\times1,2048\end{bmatrix}\times3$	$\begin{bmatrix}1\times1,512\\3\times3,512\\1\times1,2048\end{bmatrix}\times3$
	1×1	Average pool, 1000-d fc, softmax				
FLOPs		1.8×10^9	3.6×10^9	3.8×10^9	7.6×109	11.3×10^9

Region Proposal Network

The detailed architecture and data flow of the region proposal network (2) are shown in Supple. Fig. 3. For the feature maps extracted in the previous stage, the region proposal network first generates the candidate boxes (or ROI, which might contain a spark to be detected). Each point (pixel) in the feature map is called an anchor point. For each anchor point, anchor boxes are generated with different scale/size and ratio. Then it separates into two data flows. In the first data flow, each box is classified according to whether it is in the foreground (fg) or background (bg) through a 3×3 convolution to extract the characterization and an 18-channel 1×1 convolution; at the same time, the box shape is adjusted through learning to properly fit the actual object, called bounding box regression (w.r.t. a ground truth (GT) box or the spark label). In the second flow, each anchor box undergoes 3×3 convolution to extract the characterization and 36-channel 1×1 convolution for dimensionality reduction. The outputs are four coordinates (x, y, w, h) of the box and two scores for foreground (fg) and background (bg). The softmax calculates the IOU (Intersection of Union) of GT boxes (spark labels) with the anchor boxes to check whether they are fg or bg. The difference in the coordinates (x, y, w, h) between an fg

box and a GT box are calculated as targets to be learned by the regressor. The final output is a group of ROIs.

Spark Identification and Characterization

In this process, for each object proposal labeled with a box, or an ROI, the pooling layer extracts a fixed-length feature vector from the feature map. Each feature vector is fed into the sequence of fully connected layers that finally branch into two sibling output layers: one that produces softmax probability estimation over K object classes plus a catch-all “background” class, and another layer that outputs four real-valued numbers for each of the K object classes. Each set of four values encodes refined bounding-box positions for one of the K classes with calculated probability. Finally, this process outputs the detected sparks (object of probability ≥ 0.5). Then, the characterization process (1, 8) is performed to analyze the spatiotemporal parameters, including amplitude ($\Delta F/F_0$ = peak $F/F_0 - 1$), full width at half maximum (FWHM), full duration at half maximum (FDHM), rise time, and decay-to-50% time (t50) for each identified spark.

Classification of Sparks and Single Cardiomyocytes

The features from thousands of Ca^{2+} sparks in cardiomyocytes of WT or diseased groups were further used to train the logistic regression flow (9) to obtain a binary model for subsequent spark classification. Logistic regression is a typical learning method with quick implementation, good explanatory power, and easy expansion, which although called regression is actually applied to classification problems. In this study, we established a binary classification model as stated in Equation (1) that tries to differentiate between cardiomyocytes in normal and pathological states. It uses training data set x to train and build the weight vector θ, and then it maps the predicted value ($\theta^T x$) with sigmoid function $H_\theta(x)$ to binary classification. With the calculated loss, the weight vector is refined through a learning process that finalizes the weight vector. A typical refined weight vector for CPVT is shown in Equations (2.1) and (2.2) and reveals the dependency between the binary classification result and the five spark characterized properties, and Equation (2.3) is a typical vector for ISO/VEH. These equations show that $\Delta F/F_0$, *rise time*, t50, *FDHM*, *FWHM*, and *Bias* play similar roles for CPVT/WT and ISO/VEH.

$$\mathrm{h}_\theta(\mathrm{x}) = \mathrm{g}(\theta^\mathrm{T}\mathrm{x}) = \frac{1}{1+e^{-\theta^\mathrm{T} X}} \qquad (1)$$

$$\text{Vector } x = [\Delta F/F_0, \textit{rise time}, t50, \textit{FDHM}, \textit{FWHM}] + [\textit{Bias}] \tag{2.1}$$

$$\text{Weight vector } \theta = [-1.7212, 0.0552, -0.0049, 0.0300, -0.0203] + [-0.2727] \tag{2.2}$$

$$\text{Weight vector } \theta = [-1.61942, 0.0483, -0.0061, 0.0311, -0.0197] + [-0.2477] \tag{2.3}$$

All sparks in a cardiomyocyte were first classified into two indicated groups according to Equation (1), then the cardiomyocyte was classified according to the majority spark category therein.

Validation

For logistic regression used to build a binary model, statistical validation is carried out as well as the classification process. There are several approaches for this purpose, namely, general cross-validation, *K*-fold cross-validation, leave-one-out cross-validation, and random-division cross-validation. General cross-validation divides the original data randomly into two groups, one as the training set and the other as the validation set; the training set is used to train the classifier, then the validation set is used to verify the model, and the final classification accuracy is recorded as the performance index of the classifier. However, this general approach does not reach fully crossing purpose and the accuracy depends highly on the manual grouping. *K*-fold cross-validation divides the original data into *K* groups (usually equally), one as the validation set and the remaining *K*-1 groups as the training set; in this way, *K* models are obtained. The average classification accuracy of the validation sets is used as the performance index of the classifier. Leave-one-out cross-validation selects one datum for testing and everything else for training, while random-division cross-validation divides the data randomly according to a predefined ratio between training and testing. Based on the above analysis, we carried out *K*-fold, random-division, and leave-one-out cross-validation, and the measured average accuracy index was 74.9%, 75.2%, and 75.1%, respectively. This validates the proposed logistic-regression approach to building the binary model for classifying cardiomyocytes based on spark characterization properties.

Implementation and Computer Hardware

The proposed framework implementing automated Ca^{2+} spark detection, analysis, and event-based classification of single cardiomyocytes using deep learning is available for download from GITHUB as a software package (see https://github.com/BJUT-XJTU-

DigitalCell/Software). To do so, one must set up a GIT account and use GIT/LFS to clone the software package (the command is: git lfs clone https://github.com/BJUT-XJTU-DigitalCell/Software); cloning directly without LFS will generate an empty file. For convenience, the full package can also be downloaded from our group website www.xyzdigitalcell.com; go to the Released Software section and click and download "CalciumSpark_DeepLearning.zip".

The downloaded package contains three items: (i) Application_UserManual.docx which describes how to test and run this application, (ii) the application folder with executable under /CalciumAnalyzer_App/CalciumAnalyzer/CalciumAnalyzer.exe, and (iii) the test images under /TestSuite/. This software was written in Python 3.7 with all required libraries wrapped inside the application; the package does not depend on any other environment setup. However it does require the OS version to be Windows 10 or above (there is no Linux support at present), but there is no restriction on CPU/GPU versions or internal storage size. We tested this application on a Microsoft Surface computer with CPU (Intel i5, 1.6 GHz, 4 cores and 8 threads), memory (8 GB), and internal storage (128 GB), and running one test image from the TestSuite through the whole process normally took 5–10 min.

References

1. Cheng, H., L. S. Song, N. Shirokova, A. Gonzalez, E. G. Lakatta, E. Rios, and M. D. Stern. 1999. Amplitude distribution of calcium sparks in confocal images: theory and studies with an automatic detection method. Biophys J 76(2):606-617.
2. Ren, S. Q., K. M. He, R. Girshick, and J. Sun. 2017. Faster R-CNN: Towards Real-Time Object Detection with Region Proposal Networks. Ieee Transactions on Pattern Analysis and Machine Intelligence 39(6):1137-1149.
3. He, K. M., X. Y. Zhang, S. Q. Ren, and J. Sun. 2016. Deep Residual Learning for Image Recognition. 2016 Ieee Conference on Computer Vision and Pattern Recognition (Cvpr):770-778.
4. Girshick, R. 2015. Fast R-CNN. 2015 Ieee International Conference on Computer Vision (Iccv):1440-1448.
5. He, K. M., G. Gkioxari, P. Dollar, and R. Girshick. 2017. Mask R-CNN. 2017 Ieee International Conference on Computer Vision (Iccv):2980-2988.
6. Ronneberger, O., P. Fischer, and T. Brox. 2015. U-Net: Convolutional Networks for Biomedical Image Segmentation. Medical Image Computing and Computer-Assisted Intervention, Pt Iii 9351:234-241.
7. Ioffe, S., and C. Szegedy. 2015. Batch Normalization: Accelerating Deep Network Training by Reducing Internal Covariate Shift. JMLR.org.
8. Lacampagne, A., C. W. Ward, M. G. Klein, and M. F. Schneider. 1999. Time course of individual Ca2+ sparks in frog skeletal muscle recorded at high time resolution. J Gen Physiol 113(2):187-198.
9. Hosmer, D. W., S. Lemeshow, and R. X. Sturdivant. Applied logistic regression.

Supplementary Figure Legends

A

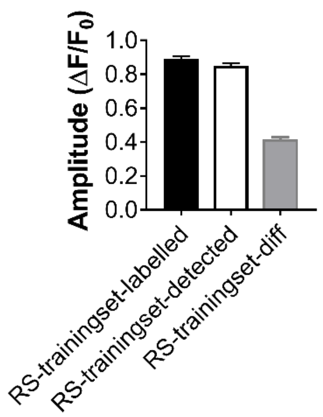

B

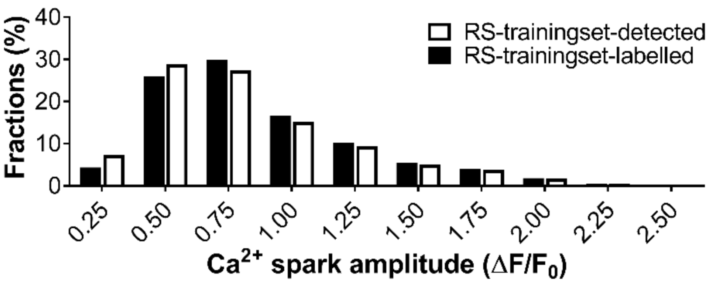

Supplementary Figure 1. Statistics (A) and distributions (B) of Ca^{2+} spark amplitude in RS cardiomyocytes in training set. The training set are pre-labelled experimental Ca^{2+} sparks that carried out with assistance of a spark characterization and detection tool set as described (1, 8). While all labelled events of high amplitude are selected automatically using the tools, the low-amplitude events are labelled manually and assessed with the tools. Thus, not all low-amplitude events have been labelled in the training set, but it can help to detected much more such low-amplitude events. In fact, using the trained model to run the training images, we can detect much more non-labelled low-amplitude events and all the labelled events.

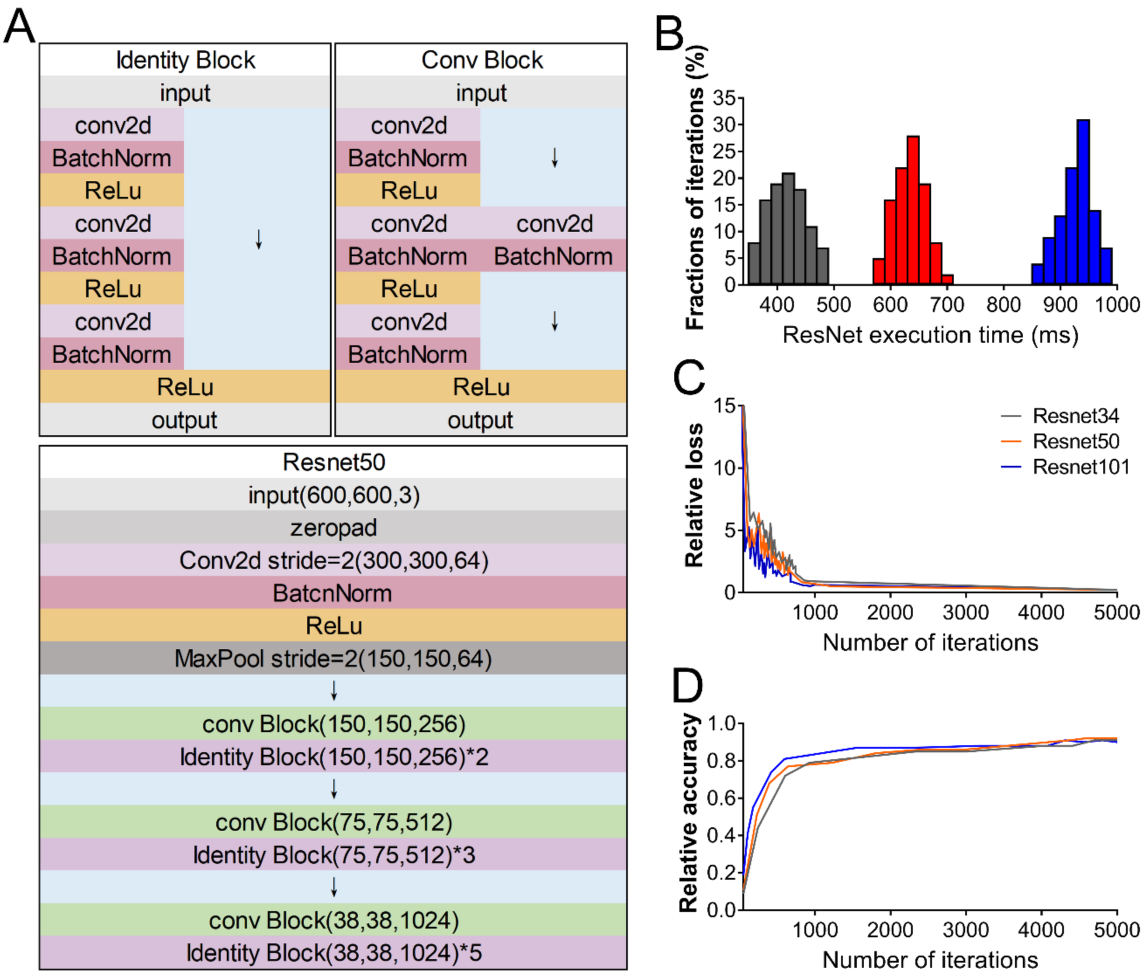

Supplementary Figure 2: ResNet structure and performance testing for 34, 50, and 101 layers. (**A**) ResNet internal structure for feature map extraction, (**B**) execution time, (**C**) relative accuracy, and (**D**) relative loss of ResNet with 34, 50, and 101 layers.

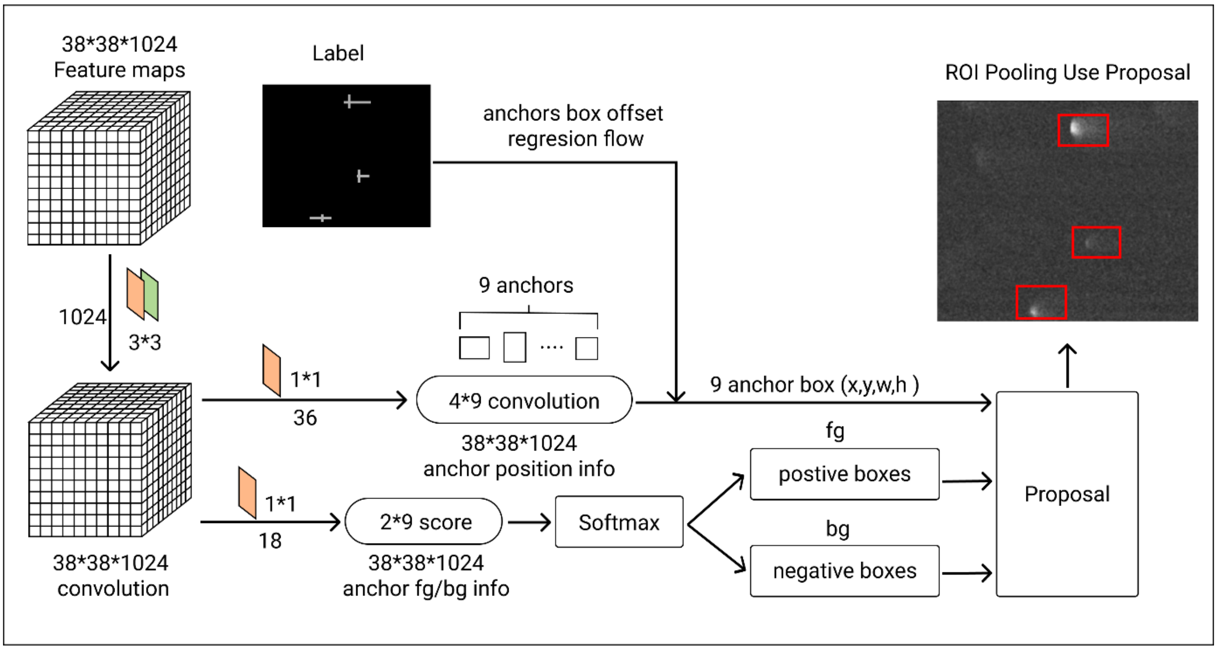

Supplementary Figure 3: Detailed architecture and data flow of region proposal network. The input of this process is the feature map extracted in the previous stage. The final output is a group of regions of interest (ROIs), which are then identified as real sparks or not.
